# CD19‐CAR engineered NK‐92 cells are sufficient to overcome NK cell resistance in B‐cell malignancies

**DOI:** 10.1111/jcmm.12810

**Published:** 2016-03-23

**Authors:** Annette Romanski, Christoph Uherek, Gesine Bug, Erhard Seifried, Hans Klingemann, Winfried S. Wels, Oliver G. Ottmann, Torsten Tonn

**Affiliations:** ^1^ Department of HematologyJ.W. Goethe University Frankfurt/MainFrankfurtGermany; ^2^ Institute for Transfusion Medicine and ImmunohematologyJ.W. Goethe University Frankfurt/MainRed Cross Blood Donor Service Baden‐Württemberg‐HessenDresdenGermany; ^3^ Georg‐Speyer‐HausInstitute for Tumor Biology and Experimental TherapyFrankfurtGermany; ^4^ Nantkwest Inc & Tufts University Medical SchoolBostonMAUSA; ^5^ Institute for Transfusion Medicine DresdenGerman Red Cross Blood Donation Service North/EastMedical Faculty Carl Gustav CarusTU DresdenDresdenGermany

**Keywords:** natural killer cell, NK‐92, CAR, αCD19, cellular immunotherapy, leukaemia

## Abstract

Many B‐cell acute and chronic leukaemias tend to be resistant to killing by natural killer (NK) cells. The introduction of chimeric antigen receptors (CAR) into T cells or NK cells could potentially overcome this resistance. Here, we extend our previous observations on the resistance of malignant lymphoblasts to NK‐92 cells, a continuously growing NK cell line, showing that anti‐CD19‐CAR (αCD19‐CAR) engineered NK‐92 cells can regain significant cytotoxicity against CD19 positive leukaemic cell lines and primary leukaemia cells that are resistant to cytolytic activity of parental NK‐92 cells. The ‘first generation’ CAR was generated from a scFv (CD19) antibody fragment, coupled to a flexible hinge region, the CD3ζ chain and a Myc‐tag and cloned into a retrovirus backbone. No difference in cytotoxic activity of NK‐92 and transduced αCD19‐CAR NK‐92 cells towards CD19 negative targets was found. However, αCD19‐CAR NK‐92 cells specifically and efficiently lysed CD19 expressing B‐precursor leukaemia cell lines as well as lymphoblasts from leukaemia patients. Since NK‐92 cells can be easily expanded to clinical grade numbers under current Good Manufactoring Practice (cGMP) conditions and its safety has been documented in several phase I clinical studies, treatment with CAR modified NK‐92 should be considered a treatment option for patients with lymphoid malignancies.

## Introduction

Acute and chronic B‐cell leukaemias can escape killing by natural killer (NK) cells. Several pathways have recently been described for resistance such as the lack of adhesion molecules on leukaemia cells 1, expression of HLA‐G 2 or the production of molecules by lymphoblasts such as MICA and MICB that bind to NK cell activating receptors like NKG2D 3, 4, 5. Many leukaemic cells also do not necessarily lose their ‘self’ MHC expression profile, a requirement for NK cells to overcome inhibitory signals through their killer cell immunoglobulin‐like receptors 6, 7.

The introduction of chimeric antigen receptors (CAR) into cytotoxic T‐ or NK cells can overcome any inhibitory signal and some significant responses in patients with advanced acute lymphoblastic leukaemia (ALL) have been reported with αCD19‐CAR engineered autologous T cells 8, 9, 10, 11, 12, 13, 14, 15.

Natural killer cells have not obtained the same level of attention as T cells for CAR engineering largely due to the fact that transfection efficiency of NK cells even with retro‐ or lentivirus is usually only 30–40% and secondly that the expansion of NK cells from peripheral blood is highly variable 16, 17. If allogeneic NK cells are used for manipulations, they need to be T cell depleted to prevent graft *versus* host disease 18, 19.

In contrast, the activated NK cell line NK‐92 can easily be expanded in culture and phase I trials have been completed showing its safety profile 20, 21, 22. Natural killer‐92 can also effectively be transfected with virus supernatant or non‐viral vectors. Even mRNA transfection using electroporation will result in at least 50% transfection efficiency 23, 24. As recently mentioned by Klingemann NK cells may be better CAR effectors than T cells for many reasons 25. Hence, NK‐92 cells are suitable alternative effector cells for CAR directed tumour cell killing.

In previous studies, retargeting of NK‐92 cells to cancer cells derived from solid tumours with a Her‐2/neu‐specific CAR resulted in efficient lysis of otherwise NK‐resistant, ErbB2/HER2‐expressing target cells *in vitro*, and enhanced tumour localization and anti‐tumoural activity *in vivo* 26, 27. In fact, the ErbB2/HER2 specific CAR NK‐92 cells have been further developed to fully meet cGMP requirements and are about to enter phase 1 clinical trials 28.

It has previously been shown that in contrast to T‐cell lymphomas, some B‐cell leukaemia cells can be resistant to NK‐92 cytotoxicity 29. Here, we provide evidence that transfection of NK‐92 with a retroviral vector encoding a first generation αCD19‐CAR can reverse resistance of cytotoxic B lineage leukaemia cells to NK‐92 killing suggesting that this strategy is suitable for the development of effective NK cell based therapeutics for the treatment of B‐cell malignancies.

## Materials and methods

### Cells and culture medium

Natural killer‐92 cell were maintained in serum free X‐VIVO 10 medium (Lonza, Cologne, Germany) containing 5% human heat‐inactivated plasma (German Red Cross Blood Donation Service Baden‐Württemberg–Hessen, Frankfurt, Germany), 1 mM L‐glutamate (Life Technologies, Darmstadt, Germany), 100 μg/ml penicillin/streptomycin (Life Technologies), supplemented with 100 U/ml IL‐2 (Chiron, Emeryville, CA, USA). Human leukaemia cell lines MOLT‐4 (T‐ALL), JKB‐1, REH, BV173, Sup‐B15, TMD‐5, TOM‐1 (B‐precursor ALLs) and K562 (CML ‐ blast crisis) were obtained from American Type Culture Collection (ATCC, Wesel, Germany) or the German Collection of Microorganisms and Cell Cultures (DSMZ, Braunschweig, Germany). TMD‐5 were obtained from Dr. Nobuo Nara (Tokyo Medical and Dental University) 30. BV173, K562, Sup‐B15, TOM‐1, MOLT‐4, REH and JKB‐1 were maintained in suspension cultures in RPMI‐1640 medium (Life Technologies) supplemented with 10% or 15% heat‐inactivated foetal calf serum (FCS; Life Technologies). TMD‐5 were cultured in MEM alpha with 15% FCS.

A number of continuously proliferating ALL cell lines (ALL‐LTCs) established from patients with ALL (ALL‐RL, ALL‐KW, ALL‐PH, ALL‐VB, ALL‐SK, ALL‐HP, ALL‐BV, ALL‐CM) were obtained from Dr. B Nijmeijer (Leiden Universitiy) and cultured as described before 31, 32.

### Patient‐derived leukaemic samples

Peripheral blood and bone marrow samples from patients with B‐cell precursor ALL were collected as part of routine diagnostic procedures. Informed consent was obtained from all patients. Collection of patient samples was approved by the ethics committee of the Goethe‐University of Frankfurt. Cells were purified on a Ficoll density gradient and cryopreserved in liquid nitrogen using RPMI‐1640 containing 10% FCS and 10% dimethylsulphoxide (Sigma‐Aldrich, Hamburg, Germany). Cryopreserved cells were thawed, washed once with Iscove medium (Biochrom KG, Berlin, Germany) plus 10% FCS, resuspended in 10 ml Cellgrow (CellGenix, Freiburg, Germany) and incubated at 37°C in 5% CO_2_ to exclude adherent cells. Mononuclear cells (PBMCs) were used directly for cytotoxicity analysis; the overall number of blast cells was consistently >90%.

### Construction of amphotropic retroviral vector and transduction of NK‐92 cells

For construction of the CD19‐specific CAR, an anti‐CD19 scFv fragment derived from plasmid pRSV/CD19‐ζ (kindly provided by C. Roessig, UKM Münster, Germany) was assembled stepwise in frame with an immunoglobulin heavy‐chain signal peptide (SP) sequence 5′ of the scFv, and sequences encoding a Myc‐tag, the hinge region of CD8α (amino acids 105–165) and CD3ζ chain 3′ of the scFv in plasmid pGEM‐1 (Promega, Mannheim, Germany). The complete CAR sequence was derived from the resulting pGEM‐1‐scFv(CD19)‐ζ construct and cloned a modified pLXSN retroviral vector yielding pL‐scFV(CD19)‐ζ‐SN 16, 27.

**Figure 1:**
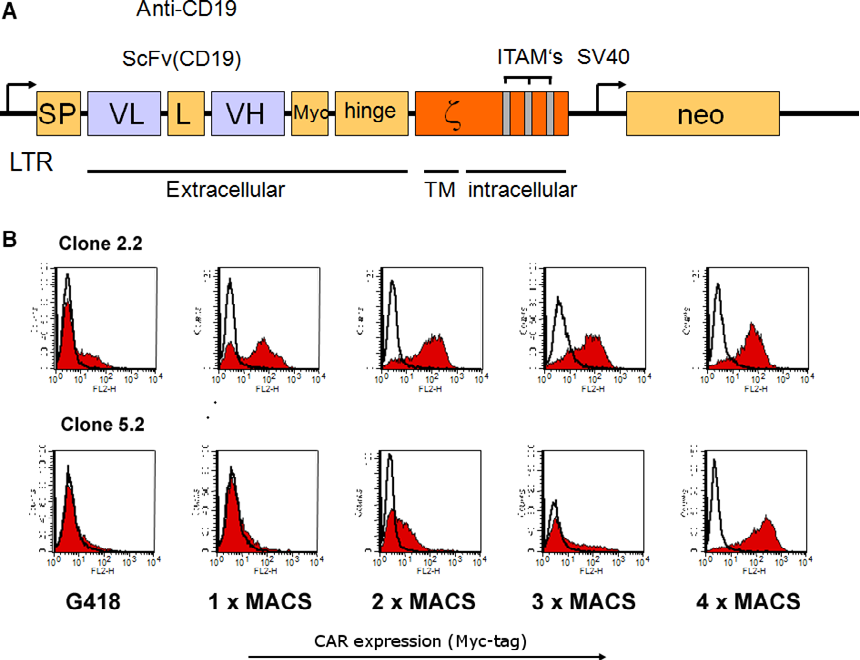
(**A**) Scheme of the retroviral construct for NK‐92 transfection consisting of specific scFv(CD19) antibody fragment, a flexible hinge region, the CD3 ζ chain and a Myc‐tag. (**B**) Transduced NK‐92 cells were selected with G418 and sorted by MACS with 1 μg anti‐Myc antibody (mAb; 9E10) per 10^6^ cells. Surface expression of the chimeric scFv(CD19)‐ζ construct was verified by FACS analysis using the Myc‐tag‐specific mAb. Open histograms indicate isotype control whereas the filled red histograms indicate the chimeric scFv(CD19)‐ζ expression. Surface expression analysis of scFv(CD19)‐ζ on NK‐92 shown here was 4 weeks after last MACS separation. G418 indicates G418 selection. 1×, 2×, 3×, 4× indicates the number of MACS separations after selection with G418.

Natural killer‐92 cells were transduced with an amphotropic retroviral vector pL‐scFV(CD19)‐ζ‐SN produced by the packaging cell line FLYA‐JET‐5 33. A schematic representation of the pL‐scFV(CD19)‐ζ‐SN construct is shown in Figure 1. The vector encodes under the control of the retroviral 5′ long terminal repeat (LTR), a fusion protein consisting of an immunoglobulin heavy‐chain leader peptide (SP), the CD19‐specific single‐chain antibody fragment (scFv(CD19)), a Myc‐tag, the hinge region of murine CD8 (amino acids 105‐165), and the murine CD3‐ζ chain 34. FLYA‐JET packaging cells 35 were transfected with pL‐scFv(CD19)‐ζ‐SN by electroporation using the Easyject Optima electroporation system (Thermo Fisher, Dreieich, Germany) with the following parameters: 20 μg of plasmid DNA per 1 × 10^6^ cells in 0.8 ml of DMEM medium (Life Technologies) in a 0.4 cm cuvette, and ‘standard’ settings according to the manufacturer's recommendations. Stable transfectants were selected for 1 week in DMEM growth medium containing 2.4 mg/ml G418 (Sigma‐Aldrich, Munich, Germany). For production of amphotropic retroviral vector, FLYA‐JET‐pL‐scFv(CD19)‐ζ‐SN cells were grown overnight in NK‐92 medium. Culture supernatant was passed through a 0.2 μm filter and incubated with NK‐92 cells in the presence of 8 μg/ml polybrene for 5 hrs at 37°C. Then NK‐92 cells were grown overnight in fresh X‐VIVO 10 medium, before G418 was added to a final concentration of 0.6 mg/ml for selection of NK‐92‐scFv(CD19)‐ζ cells (αCD19‐CAR NK‐92).

### Analysis of expression of the chimeric scFv(CD19)‐ ζ ‐ construct

Cell surface expression of scFv(CD19)‐ζ was determined by fluorescence activated cell sorter (FACS) analysis. Single‐cell suspensions (5 × 10^5^) of αCD19‐CAR NK‐92 or parental NK‐92 cells were incubated for 30 min. at 4°C with 1.5 μg of the Myc‐tag specific monoclonal antibody (mAb) 9E10 36. Cells were washed twice with PBS (Life Technoligies, Darmstadt, Germany) and then treated for another 30 min. at 4°C with a fluorescein isothiocyanate‐labelled goat anti‐mouse IgG (BD PharMingen, Heidelberg, Germany) secondary antibody. Fluorescence of cells was analysed with a FACScan (Becton Dickinson, Heidelberg, Germany).

### Enrichment of scFv(CD19)‐ ζ ‐ expressing NK‐92 cells

αCD19‐CAR NK‐92 cells expressing high levels of CAR were enriched by sorting with magnetic beads. G418‐resistant cells were incubated with mAb 9E10 (1.5 μg/5 × 10^5^ cells) and selected using goat anti‐mouse IgG MicroBeads (Miltenyi Biotec, Bergisch Gladbach, Germany) and MACS LS^+^ separation columns (Miltenyi Biotec) according to the manufacturer's instructions four times (Fig. 1).

### Cell‐mediated cytotoxicity assay

Cytotoxic activity of parental NK‐92 and αCD19‐CAR NK‐92 cells was evaluated by FACS analysis. Target cells (2 × 10^6^) were pre‐stained with the green fluorescent membrane dye PKH67‐GL (Sigma‐Aldrich) and effector cells were added to 4 × 10^4^ target cells to yield effector to target (E:T) ratios of 1:1 and 10:1. After incubation for 4 hrs, the cell mixture was centrifuged at 260 × g and stained with propidium iodide (PI, 5 μg/ml; Sigma‐Aldrich). Dead target cells were identified by simultaneous PKH67‐GL and PI ‐positive. Target cells incubated without effector cells were used to assess spontaneous cell death.

### Statistical analysis

Data are expressed as mean of triplicates ± S.D., unless stated otherwise. Data were compared by a Student's *t*‐test; *P* < 0.05 were considered to be significant.

## Results

### Cytotoxic activity of αCD19‐CAR NK‐92 cells against B‐ALL cell lines

**Figure 2:**
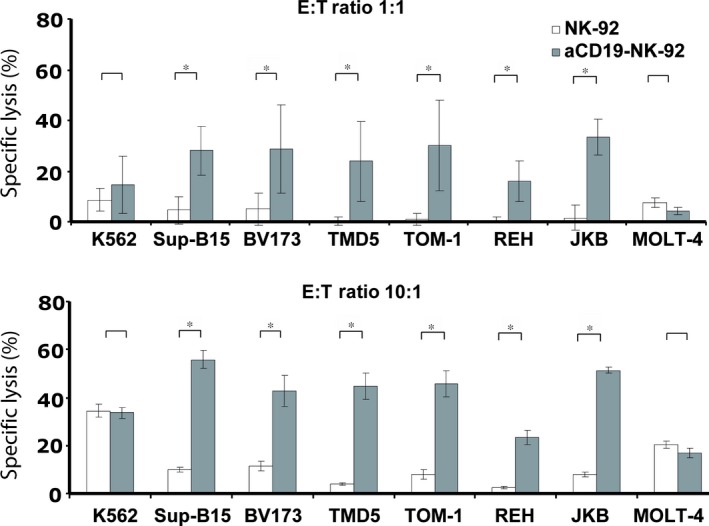
Cytotoxicity assay of αCD19‐CAR NK‐92 cells against various lymphoblastic cell lines expressing CD19. K562 and MOLT‐4 were used as CD19 negative control cells. Target cells were pre‐stained with the green fluorescent membrane dye PKH67‐GL. Cocultured effector and target cells were stained with propidium iodide, and dead target cells were quantified as double positive cells by flow cytometry. Mean values and S.D. of triplicate samples are shown (*P < 0.01, *n* = 3).

To investigate whether expression of the αCD19‐specific CAR in NK‐92 can overcome NK cell resistance of CD19 expressing lymphoblastic targets, we tested the cytotoxic activity of αCD19‐CAR NK‐92 or parental NK‐92 cells against a panel of human B‐cell leukaemia cell lines (Fig. 2: SupB15, REH, TOM‐1, TMD5, JKB‐1, BV173). By flow cytometric analysis, these cells displayed homogenous weak CD19 expression levels ranging from 52 for TOM‐1 to 272 for BV173 cells, as determined by mean channel fluorescence intensity (MFI). Lysis of those targets by parental NK‐92 cells was generally <10% at E:T ratio of 1:1 and <15% at 10:1 ratio. In contrast, lysis by αCD19‐CAR NK‐92 increased significantly to 20–38% at E:T ratios of 1:1 and 35–60% at E:T ratios of 10:1 (Fig. 2). However, killing of those target cells by αCD19‐CAR NK‐92 did not correlate with the extent of their CD19 MFI.

### Cytotoxic activity of αCD19‐CAR NK‐92 cells against B‐ALL‐LTCs

**Figure 3:**
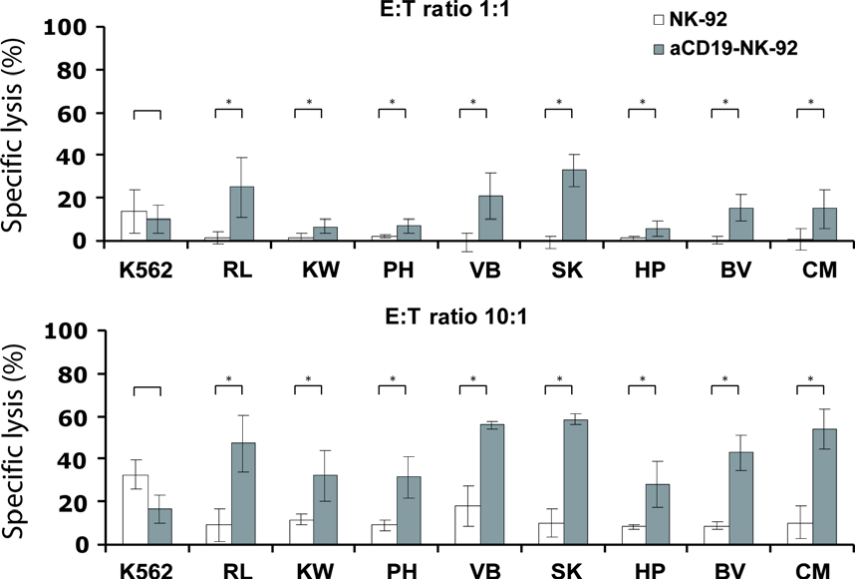
Cytotoxic activity of NK cells against primary B ALL long‐term cultures (ALL‐LTCs). Cells were analysed for expression of CD19 by flow cytometry, and used for cytotoxicity experiments. Cytotoxic activity of CD19‐specific αCD19‐CAR NK‐92 and parental NK‐92 cells was analysed as described in the legend of Figure 2. Mean values and S.D. of triplicate samples are shown (*P < 0.01, *n* = 3).

In addition to established leukaemic cell lines, we also investigated the sensitivity of patient‐derived B‐ALL‐LTCs to NK‐92 killing. As observed with cell lines, the specific lysis of these ALL‐LTCs with parental NK‐92 was 2–5% at E:T ratio of 1:1 and 5–12% at E:T ratio of 10:1. In contrast, specific lysis of the same targets with αCD19‐CAR NK‐92 as effector increased significantly to 10–30% at E:T ratio of 1:1 and 30‐60% at E:T ratio of 10:1 (Fig. 3).

### αCD19‐CAR NK‐92 cells kill NK‐resistant primary B lineage ALL cells

**Figure 4:**
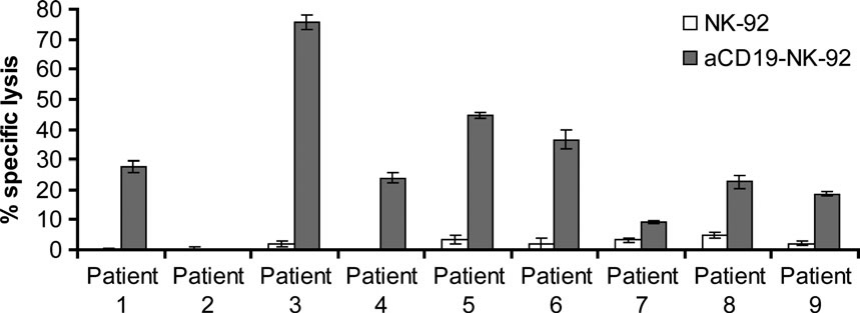
Cytotoxic activity of NK cells against primary B lineage ALL cells that were obtained from routine peripheral blood samples of untreated patients at diagnosis or at relapse. Mononuclear cells were enriched by density gradient centrifugation, analysed for expression of CD19 by flow cytometry, and used for cytotoxicity experiments. Cytotoxic activity of CD19‐specific αCD19‐CAR NK‐92 and parental NK‐92 cells was analysed as described in the legend of Figure 2. Mean values and S.E. of triplicate samples are shown (*n* = 1).

**Figure 5:**
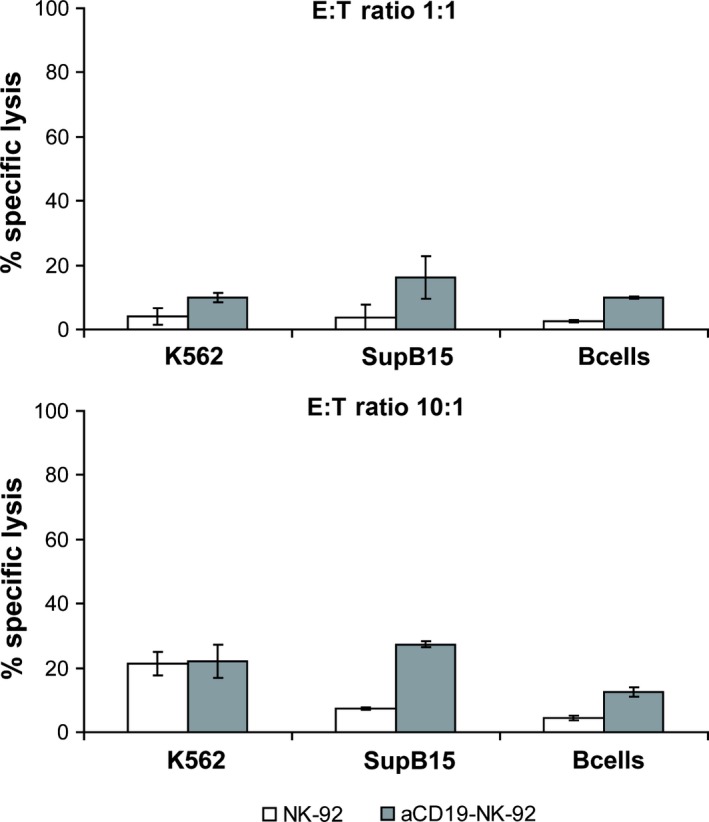
Cytotoxic activity of NK cells against B cells of healthy donors (mean of two healthy donors) in comparison to K562 (negative control) and SupB15 (positive control). Cytotoxic activity of CD19‐specific αCD19‐CAR NK‐92 and parental NK‐92 cells was analysed as described in the legend of Figure 2. Mean values and S.D. of triplicate samples are shown.

To investigate the cytotoxic activity of αCD19‐CAR NK‐92 cells against primary B lineage leukaemia, mononuclear cells were isolated from blood of patients with ALL who were either newly diagnosed or were at relapse and had over 90% leukaemic blast cells in peripheral blood. CD19 expression on the cell surface of those lymphoblasts, showed a range from 52% to 93%. Even at high E:T ratios of 10:1, primary ALL cells were not or only marginally sensitive to parental NK‐92 (1–6% specific lysis). Again, NK cell resistance could be overcome by αCD19‐CAR NK‐92 resulting in markedly enhanced killing of B‐ALL leukaemia in 8/9 patients (Fig. 4). From our data, we cannot conclude that there is a linear correlation of CD19 expression with killing by αCD19‐CAR NK‐92. However, since patient 2, whose leukaemia blasts showed resistance towards αCD19‐CAR NK‐92 did not show any CD19 surface expression, it seems likely that a threshold of CD19 receptor expression is necessary to achieve αCD19‐CAR NK‐92 mediated killing. As a control, B cells isolated from a healthy donor were co‐incubated with parental NK‐92 and αCD19‐CAR NK‐92 as effectors. NK‐92 parental cells showed very low lysis of healthy CD19 positive B cells (6%) at E:T ratio of 10:1, which was increased with αCD19‐CAR NK‐92 as effector cells (12% killing at the same E:T ratio; Fig. 5) indicating that killing is particularly enhanced in CD19 positive malignant cells.

## Discussion

The continuously growing human NK cell line NK‐92 is broadly cytotoxic against a spectrum of malignant cells 37, 38, 39. It has been shown to prolong survival in immunocompromised mouse models xenotransplanted with various human cancers 38, 39. In addition, NK‐92 cells have been administered to over 40 patients with advanced cancers as part of phase I trials. In fact these studies have proven the safety of repeated NK‐92 infusions with no significant adverse events seen in patients. The parental unmodified NK‐92 cell line, which does not express any CAR or ‘costimulatory’ molecules yielded remarkable anti‐tumour responses in some patients and therefore can be considered as a suitable vehicle to translate tumour retargeted approaches into the clinic 20, 21, 22.

The parental cell line, however, does not consistently kill lymphoid blasts 29, 40. Here, we show that cytotoxicity of NK‐92 cells against lymphoblastic target cells, including cell lines, ALL‐LTCs and primary ALL cells is effectively restored after their transfection with a CAR against CD19. This approach was originally developed to bypass MHC‐restriction in genetically modified T cells, and has been investigated in this setting for a number of surface antigens expressed on cancer cells or viruses 27, 41, 42, 43. Several phase I studies with CD19‐CAR modified T cells have shown complete and partial remissions 12, 44, 45. There can however be serious adverse events with CAR‐T‐cell infusions related to massive cytokine release as the lymphocytes expand in patients.

Chimeric antigen receptor modified NK cells have not been explored clinically. This is largely due to the fact that blood NK cells cannot be expanded as predictably as T cells and the transfection efficiency, even with optimized retro‐ or lentivirus, is generally <50% 23. On the other hand, NK‐92 cells have consistently high transfection efficiency even with mRNA 16. To date, NK‐92 cells have been armed with CAR that recognize specific ligands on target cells such as ErbB2/HER2 27, CD19 46, 47, CD20 48, CD38 49, GD2 50, EpCAM 51, EBNA 52 and CS1 53. Those CAR modified NK‐92 cells have shown consistent anti‐tumour activity *in vitro* and in SCID mouse models of human cancers 27, 40, 46, 47, 48, 49, 50, 51, 52, 53.

The objective of our current study was to investigate whether a first generation anti‐CD19 CAR would successfully kill otherwise NK‐resistant lymphoblastic leukaemia cells. The rationale for using a first generation CAR construct, lacking costimulatory motifs, such as CD28 or 4‐1BB for NK‐92 are as follows: NK‐92 already express an array of activating receptors and are therefore independent on costimulatory molecules; further NK‐92 cells are short‐living cells and are not expected to expand in the patient's circulation which obviates the need for an accessory molecule such as 4‐1BB 21.

We have previously shown that some malignant lymphoid cells, especially when they are of T‐cell origin, are killed by NK‐92 only after longer exposure of over 20 hrs suggesting additional killing mechanisms than the more rapidly lytic molecules perforin and granzyme 29. Still some lymphoid cell lines remained resistant. Here, we show that resistance in those target cells, even in short‐term cytotoxicity assays, could be restored after transfection of NK‐92 with the αCD19‐CAR. Retroviral transduction and expression of the αCD19‐CAR did not alter the intrinsic cytotoxic activity of NK‐92 against CD19 negative targets and expression profile of surface markers was not affected by CD19 CAR expression.

Primary cells from patients with ALL, which displayed little or no sensitivity to NK‐92 mediated lysis, showed markedly enhanced sensitivity to αCD19‐NK‐92. Similar results were reported for donor‐derived primary NK cells targeted to CD19 46. However, target cell killing of blood NK cells was dependent on a costimulatory 4‐1BB domain included in the CAR construct which is not required for NK‐92 activity. In addition, isolation and expansion of primary NK cells is labour‐intensive and generally yields donor dependent highly variable cell numbers. NK‐92 on the other hand grow continously and predictably and are easy to transfect making it an ‘off the shelf’ cell therapy product for immuno‐engineering.

Recent clinical trials of CAR‐T cells directed towards CD19 have shown sustained complete responses in patients with ALL and CLL 10, 54, 55, 56. Although some studies have suggested that there is a correlation between the continuous presence of CAR‐T cells in the circulation and their anti‐tumour effect, this is not a consistent observation. NK cells and NK‐92 are not expected to have a long life span in the body and would have to be infused repeatedly. However, short‐lived CAR engineered immune cells clearly have advantages as potential side effect should be less and also become more manageable. For example, patients who receive CD19‐CAR treatment develop a profound immunoglobulin deficiency since normal B cells are also targeted by CD19‐CAR, requiring frequent immunoglobulin infusions 57, 58. Since some patients can develop antigen escape over time, the persistence of CD19 CAR T cells would perpetuate the immunoglobulin deficiency without any anti‐leukaemia/lymphoma benefit, which is not a concern with CAR transfected NK‐92 cells that have a limited life span in the circulation. Any potential disadvantage of short‐lived NK‐92 cells with respect to anti‐tumour effects could be mitigated by more frequent infusions.

In summary, the highly cytotoxic cell line NK‐92 allows to generate an ‘off the shelf’ tumour specific CAR expressing cell product that is, readily available without prior need for collection of patient cells or T‐cell depletion. Furthermore, NK‐92 infusions are expected to be less burdened with severe side effects (such as cytokine release syndrome). The results presented here show that effective killing of malignant lymphoid targets can be accomplished with a first generation CAR.

## Conflicts of interest

Hans Klingemann is cofounder and equity holder of Nantkwest Inc., all other authors did not indicate any conflict of interest.

## References

[1] Fiore E , Fusco C , Romero P , *et al* Matrix metalloproteinase 9 (MMP‐9/gelatinase B) proteolytically cleaves ICAM‐1 and participates in tumor cell resistance to natural killer cell‐mediated cytotoxicity. Oncogene. 2002; 21: 5213–23. 12149643 10.1038/sj.onc.1205684

[2] Maki G , Hayes GM , Naji A , *et al* NK resistance of tumor cells from multiple myeloma and chronic lymphocytic leukemia patients: implication of HLA‐G. Leukemia. 2008; 22: 998–1006. 18288133 10.1038/leu.2008.15

[3] Fernandez‐Messina L , Reyburn HT , Vales‐Gomez M . Human NKG2D‐ligands: cell biology strategies to ensure immune recognition. Front Immunol. 2012; 3: 299. 23056001 10.3389/fimmu.2012.00299 PMC3457034

[4] Spear P , Wu MR , Sentman ML , *et al* NKG2D ligands as therapeutic targets. Cancer Immun. 2013; 13: 8. 23833565 PMC3700746

[5] Yang F , Shao Y , Yang F , *et al* Valproic acid upregulates NKG2D ligand expression and enhances susceptibility of human renal carcinoma cells to NK cell‐mediated cytotoxicity. Arch Med Sci. 2013; 9: 323–31. 23671445 10.5114/aoms.2013.34413 PMC3648824

[6] Lanier LL . Missing self, NK cells, and the white album. J Immunol. 2005; 174: 6565. 15905491 10.4049/jimmunol.174.11.6565

[7] Long EO . Regulation of immune responses through inhibitory receptors. Annu Rev Immunol. 1999; 17: 875–904. 10358776 10.1146/annurev.immunol.17.1.875

[8] De Oliveira SN , Ryan C , Giannoni F , *et al* Modification of hematopoietic stem/progenitor cells with CD19‐specific chimeric antigen receptors as a novel approach for cancer immunotherapy. Hum Gene Ther. 2013; 24: 824–39. 23978226 10.1089/hum.2012.202 PMC3787487

[9] Almasbak H , Walseng E , Kristian A , *et al* Inclusion of an IgG1‐Fc spacer abrogates efficacy of CD19 CAR T cells in a xenograft mouse model. Gene Ther. 2015; 22: 391–403. 25652098 10.1038/gt.2015.4

[10] Ghorashian S , Pule M , Amrolia P . CD19 chimeric antigen receptor T cell therapy for haematological malignancies. Br J Haematol. 2015; 169: 463–78. 25753571 10.1111/bjh.13340

[11] Gill S , June CH . Going viral: chimeric antigen receptor T‐cell therapy for hematological malignancies. Immunol Rev. 2015; 263: 68–89. 25510272 10.1111/imr.12243

[12] Kebriaei P , Huls H , Jena B , *et al* Infusing CD19‐directed T cells to augment disease control in patients undergoing autologous hematopoietic stem‐cell transplantation for advanced B‐lymphoid malignancies. Hum Gene Ther. 2012; 23: 444–50. 22107246 10.1089/hum.2011.167 PMC3360496

[13] Klein SC , Boer LH , de Weger RA , *et al* Release of cytokines and soluble cell surface molecules by PBMC after activation with the bispecific antibody CD3 x CD19. Scand J Immunol. 1997; 46: 452–8. 9393627 10.1046/j.1365-3083.1997.d01-151.x

[14] Kochenderfer JN , Dudley ME , Carpenter RO , *et al* Donor‐derived CD19‐targeted T cells cause regression of malignancy persisting after allogeneic hematopoietic stem cell transplantation. Blood. 2013; 122: 4129–39. 24055823 10.1182/blood-2013-08-519413 PMC3862276

[15] Roessig C , Scherer SP , Baer A , *et al* Targeting CD19 with genetically modified EBV‐specific human T lymphocytes. Ann Hematol. 2002; 81: S42–3. 12611072

[16] Boissel L , Betancur M , Wels WS , *et al* Transfection with mRNA for CD19 specific chimeric antigen receptor restores NK cell mediated killing of CLL cells. Leuk Res. 2009; 33: 1255–9. 19147228 10.1016/j.leukres.2008.11.024 PMC3047414

[17] Sutlu T , Nystrom S , Gilljam M , *et al* Inhibition of intracellular antiviral defense mechanisms augments lentiviral transduction of human natural killer cells: implications for gene therapy. Hum Gene Ther. 2012; 23: 1090–100. 22779406 10.1089/hum.2012.080 PMC3472531

[18] Lapteva N , Durett AG , Sun J , *et al* Large‐scale *ex vivo* expansion and characterization of natural killer cells for clinical applications. Cytotherapy. 2012; 14: 1131–43. 22900959 10.3109/14653249.2012.700767 PMC4787300

[19] Siegler U , Meyer‐Monard S , Jorger S , *et al* Good manufacturing practice‐compliant cell sorting and large‐scale expansion of single KIR‐positive alloreactive human natural killer cells for multiple infusions to leukemia patients. Cytotherapy. 2010; 12: 750–63. 20491532 10.3109/14653241003786155

[20] Arai S , Meagher R , Swearingen M , *et al* Infusion of the allogeneic cell line NK‐92 in patients with advanced renal cell cancer or melanoma: a phase I trial. Cytotherapy. 2008; 10: 625–32. 18836917 10.1080/14653240802301872

[21] Tonn T , Becker S , Esser R , *et al* Cellular immunotherapy of malignancies using the clonal natural killer cell line NK‐92. J Hematother Stem Cell Res. 2001; 10: 535–44. 11522236 10.1089/15258160152509145

[22] Tonn T , Schwabe D , Klingemann HG , *et al* Treatment of patients with advanced cancer with the natural killer cell line NK‐92. Cytotherapy. 2013; 15: 1563–70. 24094496 10.1016/j.jcyt.2013.06.017

[23] Boissel L , Betancur M , Lu W , *et al* Comparison of mRNA and lentiviral based transfection of natural killer cells with chimeric antigen receptors recognizing lymphoid antigens. Leuk Lymphoma. 2012; 53: 958–65. 22023526 10.3109/10428194.2011.634048 PMC3491067

[24] Lehner M , Gotz G , Proff J , *et al* Redirecting T cells to Ewing's sarcoma family of tumors by a chimeric NKG2D receptor expressed by lentiviral transduction or mRNA transfection. PLoS ONE. 2012; 7: e31210. 22355347 10.1371/journal.pone.0031210 PMC3280271

[25] Klingemann H . Are natural killer cells superior CAR drivers? Oncoimmunology. 2014; 3: e28147. 25340009 10.4161/onci.28147 PMC4203506

[26] Daldrup‐Link HE , Meier R , Rudelius M , *et al* *In vivo* tracking of genetically engineered, anti‐HER2/neu directed natural killer cells to HER2/neu positive mammary tumors with magnetic resonance imaging. Eur Radiol. 2005; 15: 4–13. 15616814 10.1007/s00330-004-2526-7

[27] Uherek C , Tonn T , Uherek B , *et al* Retargeting of natural killer‐cell cytolytic activity to ErbB2‐expressing cancer cells results in efficient and selective tumor cell destruction. Blood. 2002; 100: 1265–73. 12149207

[28] Schonfeld K , Sahm C , Zhang C , *et al* Selective inhibition of tumor growth by clonal NK cells expressing an ErbB2/HER2‐specific chimeric antigen receptor. Mol Ther. 2015; 23: 330–8. 25373520 10.1038/mt.2014.219 PMC4445620

[29] Romanski A , Bug G , Becker S , *et al* Mechanisms of resistance to natural killer cell‐mediated cytotoxicity in acute lymphoblastic leukemia. Exp Hematol. 2005; 33: 344–52. 15730858 10.1016/j.exphem.2004.11.006

[30] Tohda S , Sakashita C , Fukuda T , *et al* Establishment of a double Philadelphia chromosome‐positive acute lymphoblastic leukemia‐derived cell line, TMD5: effects of cytokines and differentiation inducers on growth of the cells. Leuk Res. 1999; 23: 255–61. 10071078 10.1016/s0145-2126(98)00172-6

[31] Goselink HM , van Damme J , Hiemstra PS , *et al* Colony growth of human hematopoietic progenitor cells in the absence of serum is supported by a proteinase inhibitor identified as antileukoproteinase. J Exp Med. 1996; 184: 1305–12. 8879202 10.1084/jem.184.4.1305 PMC2192846

[32] Nijmeijer BA , Szuhai K , Goselink HM , *et al* Long‐term culture of primary human lymphoblastic leukemia cells in the absence of serum or hematopoietic growth factors. Exp Hematol. 2009; 37: 376–85. 19135770 10.1016/j.exphem.2008.11.002

[33] Farag SS , Fehniger TA , Ruggeri L , *et al* Natural killer cell receptors: new biology and insights into the graft‐versus‐leukemia effect. Blood. 2002; 100: 1935–47. 12200350 10.1182/blood-2002-02-0350

[34] Tran AC , Zhang D , Byrn R , *et al* Chimeric zeta‐receptors direct human natural killer (NK) effector function to permit killing of NK‐resistant tumor cells and HIV‐infected T lymphocytes. J Immunol. 1995; 155: 1000–9. 7608531

[35] Gerstmayer B , Groner B , Wels W , *et al* Stable expression of the ecotropic retrovirus receptor in amphotropic packaging cells facilitates the transfer of recombinant vectors and enhances the yield of retroviral particles. J Virol Methods. 1999; 81: 71–5. 10488763 10.1016/s0166-0934(99)00053-1

[36] Evan GI , Lewis GK , Ramsay G , *et al* Isolation of monoclonal antibodies specific for human c‐myc proto‐oncogene product. Mol Cell Biol. 1985; 5: 3610–6. 3915782 10.1128/mcb.5.12.3610-3616.1985 PMC369192

[37] Klingemann HG , Wong E , Maki G . A cytotoxic NK‐cell line (NK‐92) for *ex vivo* purging of leukemia from blood. Biol Blood Marrow Transplant. 1996; 2: 68–75. 9118301

[38] Tam YK , Miyagawa B , Ho VC , *et al* Immunotherapy of malignant melanoma in a SCID mouse model using the highly cytotoxic natural killer cell line NK‐92. J Hematother. 1999; 8: 281–90. 10417052 10.1089/106161299320316

[39] Yan Y , Steinherz P , Klingemann HG , *et al* Antileukemia activity of a natural killer cell line against human leukemias. Clin Cancer Res. 1998; 4: 2859–68. 9829753

[40] Boissel L , Betancur‐Boissel M , Lu W , *et al* Retargeting NK‐92 cells by means of CD19‐ and CD20‐specific chimeric antigen receptors compares favorably with antibody‐dependent cellular cytotoxicity. Oncoimmunology. 2013; 2: e26527. 24404423 10.4161/onci.26527 PMC3881109

[41] Abken H , Hombach A , Heuser C . Immune response manipulation: recombinant immunoreceptors endow T‐cells with predefined specificity. Curr Pharm Des. 2003; 9: 1992–2001. 12871185 10.2174/1381612033454289

[42] Bitton N , Debre P , Eshhar Z , *et al* T‐bodies as antiviral agents. Curr Top Microbiol Immunol. 2001; 260: 271–300. 11443878 10.1007/978-3-662-05783-4_14

[43] Kershaw MH , Teng MW , Smyth MJ , *et al* Supernatural T cells: genetic modification of T cells for cancer therapy. Nat Rev Immunol. 2005; 5: 928–40. 16322746 10.1038/nri1729

[44] Huang G , Yu L , Cooper LJ , *et al* Genetically modified T cells targeting interleukin‐11 receptor alpha‐chain kill human osteosarcoma cells and induce the regression of established osteosarcoma lung metastases. Cancer Res. 2012; 72: 271–81. 22075555 10.1158/0008-5472.CAN-11-2778 PMC3833671

[45] Zhou X , Li J , Wang Z , *et al* Cellular immunotherapy for carcinoma using genetically modified EGFR‐specific T lymphocytes. Neoplasia. 2013; 15: 544–53. 23633926 10.1593/neo.13168 PMC3638357

[46] Imai C , Iwamoto S , Campana D . Genetic modification of primary natural killer cells overcomes inhibitory signals and induces specific killing of leukemic cells. Blood. 2005; 106: 376–83. 15755898 10.1182/blood-2004-12-4797 PMC1895123

[47] Marin V , Kakuda H , Dander E , *et al* Enhancement of the anti‐leukemic activity of cytokine induced killer cells with an anti‐CD19 chimeric receptor delivering a 4‐1BB‐zeta activating signal. Exp Hematol. 2007; 35: 1388–97. 17656004 10.1016/j.exphem.2007.05.018

[48] Muller T , Uherek C , Maki G , *et al* Expression of a CD20‐specific chimeric antigen receptor enhances cytotoxic activity of NK cells and overcomes NK‐resistance of lymphoma and leukemia cells. Cancer Immunol Immunother. 2008; 57: 411–23. 17717662 10.1007/s00262-007-0383-3 PMC11029838

[49] Mihara K , Bhattacharyya J , Kitanaka A , *et al* T‐cell immunotherapy with a chimeric receptor against CD38 is effective in eliminating myeloma cells. Leukemia. 2012; 26: 365–7. 21836610 10.1038/leu.2011.205

[50] Esser R , Muller T , Stefes D , *et al* NK cells engineered to express a GD2 ‐specific antigen receptor display built‐in ADCC‐like activity against tumour cells of neuroectodermal origin. J Cell Mol Med. 2012; 16: 569–81. 21595822 10.1111/j.1582-4934.2011.01343.x PMC3822932

[51] Leung K . DiD‐Labeled anti‐EpCAM‐directed NK‐92‐scFv(MOC31) zeta cells. Molecular Imaging and Contrast Agent Database (MICAD) [Internet] 2004. 20641757

[52] Tassev DV , Cheng M , Cheung NK . Retargeting NK92 cells using an HLA‐A2‐restricted, EBNA3C‐specific chimeric antigen receptor. Cancer Gene Ther. 2012; 19: 84–100. 21979579 10.1038/cgt.2011.66

[53] Chu J , Deng Y , Benson DM , *et al* CS1‐specific chimeric antigen receptor (CAR)‐engineered natural killer cells enhance *in vitro* and *in vivo* antitumor activity against human multiple myeloma. Leukemia. 2014; 28: 917–27. 24067492 10.1038/leu.2013.279 PMC3967004

[54] Levine BL . Performance‐enhancing drugs: design and production of redirected chimeric antigen receptor (CAR) T cells. Cancer Gene Ther. 2015; 22: 79–84. 25675873 10.1038/cgt.2015.5

[55] Maude SL , Shpall EJ , Grupp SA . Chimeric antigen receptor T‐cell therapy for ALL. Hematology Am Soc Hematol Educ Program. 2014; 2014: 559–64. 25696911 10.1182/asheducation-2014.1.559

[56] Weiland J , Elder A , Forster V , *et al* CD19: a multifunctional immunological target molecule and its implications for Blineage acute lymphoblastic leukemia. Pediatr Blood Cancer. 2015; 62(7): 1144–8. 25755168 10.1002/pbc.25462

[57] Grupp SA , Kalos M , Barrett D , *et al* Chimeric antigen receptor‐modified T cells for acute lymphoid leukemia. N Engl J Med. 2013; 368: 1509–18. 23527958 10.1056/NEJMoa1215134 PMC4058440

[58] Porter DL , Levine BL , Kalos M , *et al* Chimeric antigen receptor‐modified T cells in chronic lymphoid leukemia. N Engl J Med. 2011; 365: 725–33. 21830940 10.1056/NEJMoa1103849 PMC3387277

